# Lightweight Monocular Distance Estimation via Anisotropic Geometry Loss for Low-Light Driving Environments

**DOI:** 10.3390/s26144440

**Published:** 2026-07-13

**Authors:** Ricky Christanto, Shaou-Gang Miaou

**Affiliations:** Department of Electronic Engineering, Chung Yuan Christian University, Taoyuan 320314, Taiwan; g11202603@cycu.edu.tw

**Keywords:** monocular distance estimation, illumination-invariant vision, autonomous emergency braking, efficient GPU computing, nocturnal autonomous driving

## Abstract

**Highlights:**

**What are the main findings?**

- We introduce the Anisotropic Geometry Loss (AGL) framework, a 2.71 M-parameter monocular distance estimator that achieves >160 FPS while reducing Dark-KITTI RMSE from 11.53 m (Vanilla YOLOv10n) to 10.55 m with the optional CLAHE preprocessor—without modifying the YOLOv10n backbone.
- Joint sensitivity analysis of the loss coefficients (*λ_y_*, *λ_h_*) reveals that independent single-axis tuning misidentifies the global optimum and exposes seed-instability at apparent minima—motivating cross-seed-stable coefficient selection over greedy single-axis tuning for lightweight monocular 3D detectors.

**What are the implications of the main findings?**

- Explicit pinhole-geometry constraints, rather than heavyweight architectural depth-prediction heads, are sufficient to suppress the multi-meter monocular depth errors caused by floating bounding boxes, enabling camera-only Autonomous Emergency Braking (AEB) to demonstrate computational efficiency on desktop GPU and provide a promising foundation for future embedded deployment aligned with Euro NCAP safety protocols.
- The Far-zone (>30 m) results demonstrate that all evaluated methods converge to a similar localization accuracy, reflecting the fundamental limitation of static monocular projection rather than model-specific performance. The proposed AGL framework therefore delivers its principal benefit in the Medium zone (15–30 m), while maintaining equivalent Far-zone performance to existing techniques. This behavior is consistent with Euro NCAP monitoring-only requirements beyond 30 m and suggests that future improvements for long-range localization should incorporate dynamic camera-pose estimation and graded-terrain compensation.

**Abstract:**

Robust monocular distance estimation under varying illumination conditions is critical for autonomous driving safety. While state-of-the-art monocular 3D detection models achieve high accuracy in daylight conditions, they rely on computationally heavy architectures and degrade significantly in low-light environments. Lightweight 2D detectors (e.g., YOLO variants) offer real-time performance but lack the geometric constraints required for accurate depth estimation. To address this limitation, we propose the Anisotropic Geometry Loss (AGL) framework. This lightweight framework enforces ground-plane consistency through an anisotropic bottom-edge constraint derived from the pinhole camera model. In addition, a luminance-channel contrast enhancement module (CLAHE) is applied at inference to improve low-light visibility. Experimental results on the Dark-KITTI dataset show that the proposed method achieves an RMSE of 10.91 ± 0.68 m, improving over YOLOv10n (11.53 ± 0.56 m) and YOLOv26n (11.99 ± 0.58 m), while maintaining a 2.71 M-parameter footprint and real-time inference (>160 FPS). With CLAHE, RMSE is further reduced to 10.55 ± 0.72 m. Stratified by kinematic safety zone, the proposed method achieves 2.42 ± 0.03 m in the Near range (0–15 m), 5.94 ± 0.19 m in the Medium range (15–30 m), and 17.41 ± 1.25 m in the Far range (>30 m), corresponding to Euro NCAP AEB (Autonomous Emergency Braking) stopping distances. AGL provides its largest measurable accuracy improvement in the medium-distance range while maintaining comparable performance in the far-distance range. A complementary luminance-channel CLAHE preprocessor recovers bottom-edge gradients in synthetic and real low-light frames; zero-shot generalization is qualitatively corroborated on the ExDark dataset. These results demonstrate that explicit geometric constraints provide an effective and efficient solution for robust cross-illumination resistance in monocular distance estimation. The framework also shows practical potential for camera-only AEB systems deployed on edge-computing platforms aligned with Euro NCAP safety protocols.

## 1. Introduction

The transition of autonomous vehicles (AVs) from highly controlled operational design domains to unstructured, real-world environments requires perceptual systems that remain robust under severe visual degradation [1]. Nighttime driving remains disproportionately dangerous, as standard camera sensors suffer from low signal-to-noise ratios, motion blur, and headlight glare [2]. In driving environments, illumination degradation and exposure imbalance can significantly affect scene perception and safety-related decision making. Previous studies have investigated exposure correction techniques specifically for driving scenarios to improve visual quality under challenging lighting conditions [3].

Our work targets lightweight computational efficiency on desktop GPU and provides a promising foundation for future embedded deployment. A sub-3 M-parameter detector operating under real-time AEB latency budgets—where heavyweight monocular 3D architectures are not viable design candidates.

**Figure 1:**
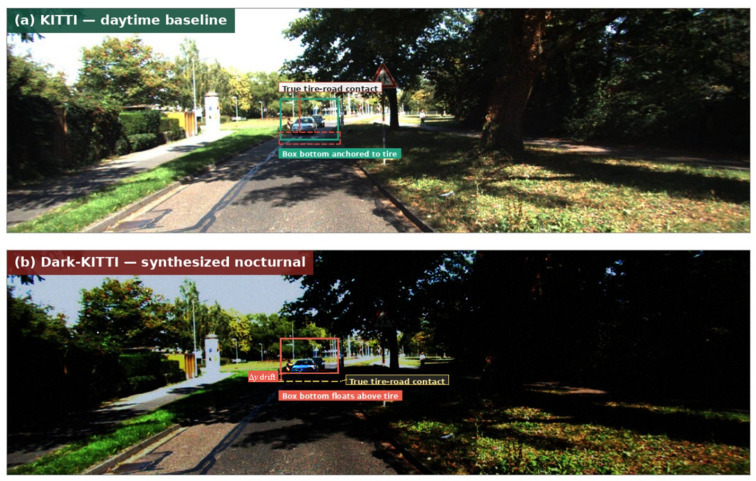
The Multi-Illumination Domain Gap. (**a**) KITTI daytime baseline: under high ambient illumination, the tire-road contact interface is sharply delineated (coral dashed region), and the predicted bounding box (teal) is correctly grounded to the true ground-contact pixel, enabling stable monocular pinhole projection. (**b**) Dark-KITTI synthesized nocturnal scene: the same tire-road interface is submerged into the elevated luminance noise floor; the predicted bounding box (coral, solid) “floats” above the true ground-truth contact (yellow dashed line) by Δ*y* pixels, producing the multi-meter depth error mathematically derived in Section 3.2.

As illustrated in Figure 1, transitioning from diurnal to nocturnal environments significantly degrades high-frequency spatial features. In the daytime baseline (top), the tire-road interface is clearly delineated, allowing for stable geometric anchoring. However, in the nocturnal domain (bottom), low-light noise and reduced dynamic range cause the vehicle’s lower boundary to submerge into the road’s luminance floor, resulting in the so-called ‘floating bounding box’ phenomenon.

Monocular distance estimation relies on accurate extraction of the tire-road interface for geometric anchoring. As shown in the transition from KITTI (day) to the Dark-KITTI (night) variant, low-light conditions and an elevated sensor noise floor result in the “submergence” of high-frequency spatial features. This visual degradation leads to vertical bounding box drift, which, as mathematically proven in Section 3.2, results in hyperbolic (inverse-square) distance estimation errors.

While LiDAR provides illumination-invariant 3D geometry, its high cost and hardware complexity restrict its use in mass-market edge devices, driving the need for purely monocular camera solutions [4].

Recent SOTA monocular 3D detectors achieve high spatial accuracy in daylight conditions [5,6,7,8]. However, these models inherently rely on dense geometric feature extraction [9,10] and large model capacity, limiting their deployment on edge devices. Moreover, their performance degrades significantly in low-light scenarios due to the loss of high-frequency visual features [2]. In contrast, lightweight 2D detectors [11,12,13] provide efficient inference but lack geometric awareness. Their reliance on isotropic IoU-based loss functions [14] leads to vertical bounding-box drift, resulting in large depth estimation errors under the pinhole camera model [15].

To address these limitations, we propose the Anisotropic Geometry Loss (AGL) framework, a lightweight system for robust monocular distance estimation under cross-illumination conditions. The framework enforces geometric consistency through an anisotropic loss term—denoted *L_y_*_tall_—applied to the bounding-box bottom edge, and is further enhanced with a lightweight contrast normalization module for low-light environments. Throughout this paper, we use AGL to denote the trained detection framework (YOLOv10n + *L_y_*_tall_ loss + decoupled head + safety clamp), *L_y_*_tall_ to denote the loss term itself (defined in Section 3.3.1, combining Equations (5) and (6)), and AGL + CLAHE to denote the framework with the optional nocturnal preprocessor applied at inference time. The contributions of this work are summarized as follows:

1. We derive an inverse-square sensitivity analysis of the pinhole projection that mathematically motivates anisotropic bottom-edge weighting for monocular distance estimation, providing a principled alternative to isotropic IoU optimization.
2. We propose the *L_y_*_tall_, a lightweight two-term penalty (*L*_position_ + *L*_height_) that integrates into the YOLOv10n decoupled head without modifying the backbone, preserving real-time inference at >160 FPS on a 2.71 M-parameter footprint.
3. We introduce a luminance-channel CLAHE [16] preprocessor that recovers tire-road micro-contrast under nocturnal degradation [17], with asymmetric utility validated on synthetic Dark-KITTI and qualitatively corroborated on real ExDark imagery.
4. We further show that the two *L_y_*_tall_ coefficients, *λ_y_* and *λ_h_*, which respectively govern vertical geometric alignment and object-scale consistency, exhibit strongly coupled optimization behavior rather than independently additive effects. This finding exposes a key limitation of conventional single-axis hyperparameter tuning. The apparent optimum of one coefficient shifts with the value of the other, potentially leading to misleading performance interpretations. To address this issue, we conduct a comprehensive joint sensitivity analysis to identify the true coupled optimum and establish a principled, reproducible guideline for coefficient selection in lightweight monocular 3D object detection.

Within these constraints, our experiments demonstrate that the AGL framework mitigates severe nocturnal domain shifts while maintaining inference speeds above 160 frames per second on highly constrained Nano architectures [18].

## 2. Related Work

### 2.1. Monocular 3D Object Detection

Monocular vision-based distance estimation has attracted significant attention due to its low hardware cost and ease of deployment. Recent advances in monocular depth estimation have focused on improving accuracy while maintaining computational efficiency for deployment in intelligent transportation systems. A recent survey by Huang et al. highlighted lightweight architectures, self-supervised learning, and robustness under challenging environmental conditions as major research directions in autonomous driving applications [19]. Modern monocular 3D detection models aim to recover lost depth information by imposing complex geometric constraints [20]. Architectures such as GUPNet++ [5], MonoCon [8], and SMOKE [6] utilize sophisticated uncertainty prediction to achieve single-digit depth error on the standard KITTI dataset [21]. However, these models require upwards of 85 to 120 million parameters. This computational burden limits their deployment on standard AV edge hardware, and their reliance on high-fidelity visual textures makes them less robust when deployed across domains into low-light environments [22]. Therefore, we restrict the empirical comparison to lightweight 2D backbones operating at identical resolutions and with identical pinhole projections.

### 2.2. Advancements in 2D Architectures

Recent iterations of real-time object detectors, including YOLOv26 in an arXiv preprint [23], have introduced mechanisms such as Progressive Loss Balancing (ProgLoss) and Small-Target-Aware Label Assignment (STAL) to maximize 2D recall (mAP). While these innovations improve an architecture’s ability to identify distant objects, they remain limited to 2D pixel space. Because they optimize for isotropic region overlap (IoU), they do not inherently address the monocular depth-projection failure caused by floating bounding boxes. Therefore, improving 2D IoU alone does not resolve 3D spatial errors [24]. Furthermore, recent architectures have heavily integrated attention mechanisms, ranging from Transformer-based architectures like DETR to attention-augmented detectors such as YOLOv12. Sui et al. developed a fusion-improved transformer framework for efficient monocular depth estimation [25], while Xia et al. demonstrated the effectiveness of pyramid transformers combined with multi-scale feature fusion for dense depth prediction [26]. While global self-attention improves occlusion handling by aggregating contextual image features, the localization heads of these architectures remain tethered to isotropic overlap metrics (e.g., GIoU or L1 loss). Consequently, attention-based models do not inherently encode 3D ground-plane awareness. They permit vertical bounding-box drift, which can degrade monocular pinhole distance projections.

### 2.3. Low-Light Perception

Low-light image enhancement remains an important research direction for improving perception reliability under adverse illumination conditions. Recent studies have utilized dynamic illuminance adjustment strategies to enhance image visibility and exposure consistency [27]. Efforts to improve nighttime perception typically fall into either sensor fusion or synthetic data generation [28]. Sensor fusion combines degraded camera feeds with LiDAR or Radar [29], violating the constraints of low-cost monocular systems [30]. Alternatively, Generative Adversarial Networks (GANs) [31] convert daytime images into synthetic nighttime data for training. While this augments datasets, it introduces generative hallucinations and does not mathematically fix the geometric failure of the bounding box itself [32]. Zhang and Lee introduced an exposure-aware diffusion framework to improve depth estimation robustness under adverse lighting conditions, demonstrating the continued importance of illumination-aware perception methods for autonomous driving systems [33].

Existing monocular 3D detection methods typically recover depth through dense feature extraction, keypoint estimation, uncertainty modeling, or attention-based contextual aggregation. While these approaches improve spatial reasoning under favorable illumination, they remain computationally intensive and continue to optimize localization primarily through isotropic overlap objectives. Consequently, they do not explicitly constrain the geometric stability of the tire–road contact boundary that governs monocular pinhole projection. In contrast, the proposed Anisotropic Geometry Loss (AGL) introduces an explicit bottom-edge-constrained anisotropic penalty derived from the inverse-square sensitivity of the pinhole camera model. Rather than increasing model capacity or attention complexity, AGL directly stabilizes the physically meaningful projection denominator responsible for depth estimation.

Geometry-aware learning has become an active research direction in monocular depth estimation. Xiong et al. incorporated geometric constraints into a self-supervised learning framework to improve depth consistency and estimation accuracy [34]. Such findings suggest that explicitly modeling geometric relationships can substantially improve depth prediction performance. Unlike these approaches, the proposed AGL focuses on directional distance estimation errors under low-light driving conditions.

Recent lightweight low-light image enhancement methods have demonstrated promising performance through channel-prior-guided enhancement and gamma correction mechanisms [35]. Such enhancement techniques can improve image visibility and feature representation, thereby benefiting downstream perception tasks such as object detection and distance estimation in low-light environments. Nevertheless, these approaches still require a dedicated learning-based inference stage. In contrast, CLAHE provides a simple and practical preprocessing solution without additional model parameters, making it suitable for resource-constrained deployment while improving local contrast prior to object detection and distance estimation. The framework is specifically designed for lightweight edge-scale detectors by demonstrating computational efficiency on a desktop GPU, enabling real-time cross-illumination distance estimation at >160 FPS while preserving a compact 2.71 M-parameter footprint.

## 3. Methodology

**Figure 2:**
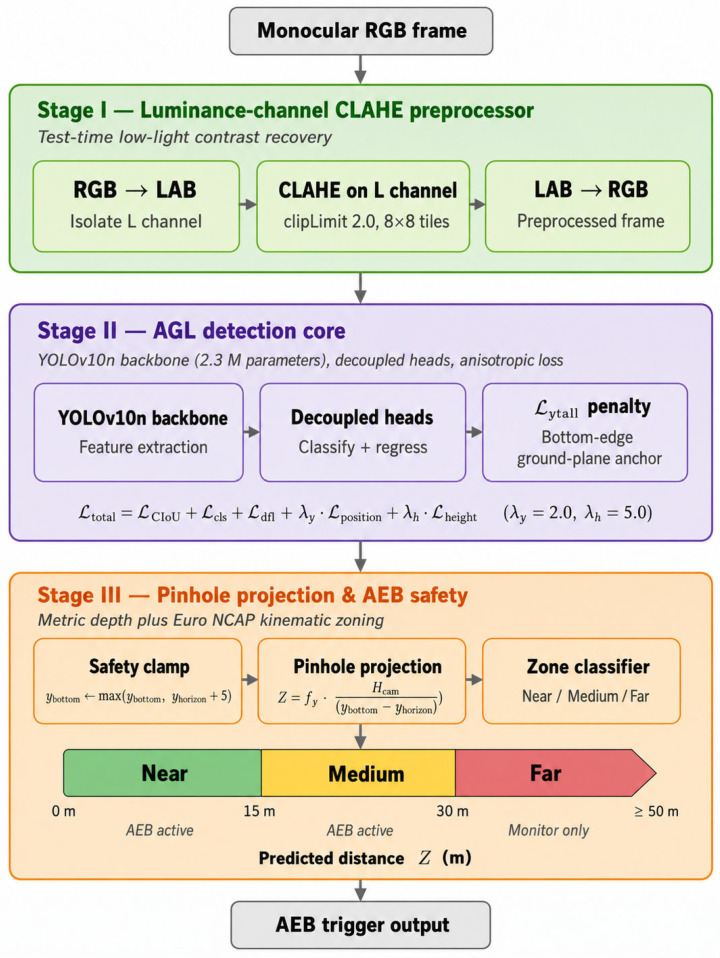
Overview of the proposed AGL system architecture, partitioned into three stages: CLAHE preprocessor (Stage I), AGL detection core (Stage II), and pinhole projection + AEB safety logic (Stage III).

Figure 2 illustrates the proposed hierarchical system architecture of the AGL framework. The pipeline is partitioned into three hierarchical execution stages designed for computationally efficient deployment. Stage I utilizes CLAHE to restore micro-contrast at the object’s base. Stage II employs a lightweight YOLOv10n backbone (2.3 M parameters) modified with an anisotropic *y*_tall_ regression penalty to enforce ground-plane stability. Stage III executes a pinhole camera projection and kinematic safety logic to classify objects within critical stopping distance zones (15 m/30 m) in accordance with Euro NCAP safety standards.

### 3.1. Pinhole Geometry of Monocular Distance Recovery

Monocular distance estimation is fundamentally based on the pinhole camera model—a deterministic projective mapping from 3D world coordinates to 2D image pixels. Standard 2D object detectors operate exclusively in pixel space and discard the metric depth information implicit in this mapping. The image coordinate system follows the conventional computer vision convention, where the x-axis points rightward and the y-axis points downward. The central observation underlying our framework is that, under a small set of mild assumptions, depth can be recovered in closed form from a single geometrically stable pixel feature: the bottom edge of an object’s bounding box. This section formalizes that recovery and identifies the sensitivity structure that motivates the loss design in Section 3.3.

**Figure 3:**
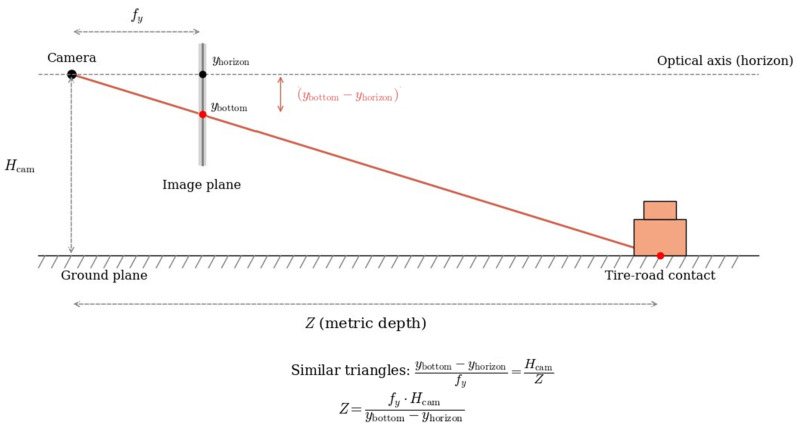
Pinhole geometry of monocular distance recovery under the flat-ground assumption. The camera is mounted at height *H*_cam_ above a planar ground surface with focal length *f_y_* and principal-point row *c_y_* = *y*_horizon_. A target object’s tire-road contact line projects to image row *y*_bottom_, and its world depth *Z* is given by Equation (2) as a hyperbolic function of the pixel offset (*y*_bottom_ − *y*_horizon_). The hyperbolic form implies that a unit-pixel error at the bottom edge translates into a metric depth error that scales as *Z*^2^, motivating the anisotropic bottom-edge penalty *L_y_*_tall_ introduced in Section 3.3.

Figure 3 illustrates the geometric setup. The camera is rigidly mounted at a fixed height *H*_cam_ above a planar ground surface, with its optical axis parallel to the ground surface (zero pitch). The image row at which the ground horizon projects, *y*_horizon_, divides the image into above-horizon and below-horizon regions. A target object whose tire-road contact line projects to image row *y*_bottom_ has its world depth *Z* fully determined by the camera intrinsics and the offset (*y*_bottom_ − *y*_horizon_), as derived below.

Formally, the projection of a world point (*X*_w_, *Y*_w_, *Z*_w_) to image pixel coordinates (*x*, *y*) is governed by the pinhole equation:

(1) $$Z_{c}\left[\begin{array}{c} x\\ y\\ 1 \end{array}\right]=\left[\begin{array}{c} f_{x}\\ 0\\ 0 \end{array}\;\;\;\;\;\;\;\;\begin{array}{c} 0\\ f_{y}\\ 0 \end{array}\;\;\;\;\;\;\;\;\begin{array}{c} c_{x}\\ c_{y}\\ 1 \end{array}\right]\left[\begin{array}{c} X_{w}\\ Y_{w}\\ Z_{w} \end{array}\right]$$

where *f_x_*, *f_y_* are the focal lengths in pixel dimensions, (*c_x_*, *c_y_*) is the principal point (the optical-center projection in image space; for KITTI, taken at the image center), and *Z*_c_ is the camera-frame depth—the *z*-component of the point’s position in camera coordinates, which serves as the homogeneous-coordinate scale factor. Under the flat-ground assumption with the camera optical axis parallel to the ground plane, *Z*_c_ coincides with the world depth *Z*_w_; we therefore adopt the unsubscripted symbol *Z* throughout the remainder of the paper.

Placing the world origin at the camera’s optical center, any point situated on the ground plane has a world *Y*-coordinate *Y*_w_ = −*H*_cam_. Substituting this constraint into the *v*-row of Equation (1) and rearranging yields a closed-form expression for the depth of a ground-contact point in terms of a single observable, *y*_bottom_:

(2) $$Z=\frac{f_{y}\;\cdot \;H_{cam}}{y_{bottom}\;-\;y_{horizon}}$$

where *y*_horizon_ is the image row at which the optical vanishing point projects (equivalent to *c_y_* under the zero-pitch assumption). Equation (2) is the geometric anchor of the entire framework: for any detected object whose bounding-box bottom edge corresponds to a point on the ground plane, the depth is determined by a single observable—the pixel offset (*y*_bottom_ − *y*_horizon_)—scaled by the two calibration constants *f_y_* and *H*_cam_. Two consequences follow directly from this mapping and motivate the loss design in Section 3.3:

- **Inverse-square sensitivity.** Differentiating Equation (2) with respect to *y*_bottom_ shows that the depth error scales as Δ*Z* ∝ *Z*^2^ · Δ*y*/(*f_y_* · *H*_cam_); a unit pixel error at the bottom edge produces a metric depth error that grows quadratically with distance. This is the formal basis for the anisotropic bottom-edge weighting in the *L_y_*_tall_ loss.
- **Bottom-edge specialization.** The recovery depends exclusively on the bounding box’s bottom edge, not its center, width, or top edge. Standard isotropic IoU-based regression treats all four box edges as equivalent, dispersing supervision across geometrically uninformative coordinates. *L_y_*_tall_ concentrates supervision on the single edge that carries the metric depth signal.

The flat-ground and zero-pitch assumptions underlying Equation (2) merit explicit treatment. On graded terrain or during dynamic vehicle motion (braking, acceleration, suspension travel), a non-zero pitch angle *ψ* introduces an additive bias to *y*_horizon_ of approximately *f_y_* · tan(*ψ*), which propagates into the depth estimate via the inverse-square sensitivity noted above. At the KITTI calibration values (*f_y_* = 721.53 px, *H*_cam_ = 1.65 m), a pitch deviation of 1° produces a horizon shift of approximately 12.6 pixels and a relative depth error of approximately 31% at *Z* = 30 m. The KITTI recording vehicle operates predominantly on level urban roads, validating the static-horizon assumption for the numerical results reported throughout this paper. Extension of the framework to graded terrain via lightweight dynamic pose estimation is reserved for future work and discussed in Section 7.

### 3.2. The Non-Linear Depth Decay and the Necessity of L_ytall_

Standard object detectors use IoU-based loss functions [14], which treat bounding-box errors isotropically. Unlike standard IoU-based loss functions that assume a linear relationship between bounding box pixel error and spatial error, the proposed *L_y_*_tall_ anisotropic penalty explicitly maps the non-linear monocular projection geometry. Taking the first derivative of the depth equation with respect to *y*_bottom_ reveals the true, non-linear hyperbola of monocular depth:

(3) $$\frac{dZ}{dy_{bottom}}=-\frac{f_{y}\cdot H_{cam}}{{\left(y_{bottom}-y_{horizon}\right)}^{2}}$$

The derivative shows that depth sensitivity increases hyperbolically (as an inverse-square function) as the bounding box prediction (*y*_bottom_) approaches the optical horizon (*y*_horizon_). A 5-pixel error at the bottom of the image (near range) results in a centimeter-scale depth error. Conversely, the same 5-pixel error near the horizon (far range) results in a multi-meter depth error of dozens of meters.

Standard 2D detectors like YOLOv10n optimize for IoU, allowing the bottom edge of the box to “float” a few pixels above the tires without penalty. While acceptable for 2D classification, the mathematical proof above demonstrates why this floating behavior causes large multi-meter distance errors. To counteract this, AGL introduces the *y*_tall_ Anisotropic Penalty, forcing the network to explicitly minimize the squared bottom-edge error (*y*_bottom_ − *y*_horizon_)^2^, ensuring the structural integrity of the denominator in our depth projection.

To prevent numerical instability (division by zero) when visual noise causes a bounding box to cross the optical horizon, we dynamically enforce a spatial safety clamp:

(4) $$y_{bottom}=max(y_{bottom},y_{horizon}+5.0)$$

At the KITTI calibration values (*f_y_* = 721.53, *H*_cam_ = 1.65 m), this +5.0 pixel buffer corresponds to a maximum detectable range of *Z* = 721.53 × 1.65/5 ≈ 238 m—well beyond the Euro NCAP’s 30 m far boundary—ensuring numerical stability without truncating any safety-relevant detection. This +5.0 pixel buffer represents the practical limit of the sensor’s far-range visibility, ensuring safe and stable computations within the Autonomous Emergency Braking (AEB) kinematic logic.

### 3.3. Hierarchical Loss Function Design

The key contribution of AGL lies in modifying the objective function used during backpropagation. Standard 2D detectors use isotropic loss functions that treat vertical and horizontal bounding-box errors equally. However, as proven in Section 3.2, monocular depth estimation is uniquely sensitive to vertical drift. To resolve this, we propose a Hierarchical Loss Architecture that integrates our custom geometric penalty into the modern decoupled head of the YOLOv10n backbone [13]. YOLOv10n was selected precisely because it eliminates Non-Maximum Suppression (NMS), guaranteeing consistent inference speeds on edge hardware without post-processing latency bottlenecks.

Furthermore, standard object detectors historically relied on shared “sibling heads” for both classification and localization. However, these objectives are inherently misaligned; classification requires translation invariance to recognize objects, while localization requires translation variance to regress the bounding box coordinates precisely [36]. Modern architectures address this via decoupled heads [37,38]. This architectural decoupling is critical to AGL, as it allows the anisotropic penalty to be applied exclusively to the bounding-box regression head. By strictly optimizing the localization head for 3D ground-plane adherence, we force the network to learn geometric constraints without compromising the classification head’s 2D recall.

#### 3.3.1. The *y*_tall_ Anisotropic Ground-Plane Penalty

We define the positional loss *L*_position_ as an anisotropic penalty that specifically targets the lower boundary of the predicted bounding box. Unlike the standard IoU loss, which optimizes the entire area of the box, *L*_position_ acts as a spatial anchor. It ensures that the predicted ground-contact point *y*_pred_bottom_ converges toward the true tire-asphalt interface *y*_target_bottom_.

We utilize the *L*_2_-norm (Squared Error) for this penalty to generate an increasingly aggressive gradient as the box “floats” away from the road surface:

*L*_position_ = (*y*_pred_bottom_ − *y*_target_bottom_)^2^ (5)

However, penalizing only the bottom-edge position is insufficient in practice, as the bottom edge is co-determined by the bounding box vertical center and its height: *y*_bottom_ = *y*_center_ + *h*/2. A network optimizing solely on Equation (5) may satisfy the constraint by inflating the predicted bounding box height while leaving the true ground-contact pixel uncovered, thereby undermining the geometric anchoring objective. To prevent this compensatory behavior, we introduce a complementary height regularization term that penalizes deviation between predicted and target bounding box heights:

*L*_height_ = (*h*_pred_ − *h*_target_)^2^ (6)

The positional term penalizes the vertical displacement of the predicted bottom edge: *L*_position_ = (*y*_pred_bottom_ − *y*_target_bottom_)^2^. The full tall penalty combines both terms: *L_y_*_tall_ = *λ_y_* · *L*_position_ + *λ_h_* · *L*_height_, where *λ_y_* and *λ_h_* are the weighting coefficients introduced in Section 3.3.2. The decomposition explicitly separates positional and scale-related constraints: *λ_y_* controls how strongly the network is pulled toward the correct bottom-edge position. At the same time, *λ_h_* prevents the network from satisfying the position constraint via height inflation. The empirical setting of these coefficients (*λ_y_* = 2.0, *λ_h_* = 5.0) is justified in Section 4.5 below.

#### 3.3.2. Total Multi-Task Objective Function

To ensure that the network maintains high 2D detection performance while gaining 3D awareness, the *L_y_*_tall_ penalty is integrated into a multi-task loss formulation. The total objective function *L*_total_ is defined as follows:

*L*_total_ = *λ*_box_*L*_CIoU_ + *λ*_cls_*L*_cls_
*+ λ*_dfl_*L*_dfl_
*+ λ_y_L_y_*_tall_ (7)

where each *λ* is a scalar weighting hyperparameter that balances the constituent loss terms. *L*_CIoU_ (Complete-IoU Loss) optimizes the overlap, aspect ratio, and center-distance of the bounding box. *L*_cls_ (Classification Loss) uses Binary Cross-Entropy to ensure correct category identification (e.g., Car vs. Pedestrian). *L*_dfl_ (Distribution Focal Loss) optimizes the probability distribution of the box boundaries for sub-pixel accuracy. *L_y_*_tall_ (AGL Innovation) enforces the geometric ground-plane constraint, implemented as the two-term penalty *L_y_*_tall_ = *λ_y_* · *L*_position_ + *λ_h_* · *L*_height_ defined in Section 3.3.1, with *λ_y_* = 2.0 and *λ_h_* = 5.0.

The coefficient *λ_y_* is a critical hyperparameter that balances the geometric constraint against traditional 2D detection goals. If *λ_y_* is too low, the model defaults to standard 2D behavior with high depth error; if too high, the model may experience bounding box collapse, where it prioritizes the bottom edge so much that it loses track of the object’s height and width. As empirically demonstrated in Section 5.4, increasing *λ_y_* beyond the optimal regime results in measurable degradation in RMSE, providing quantitative evidence of the trade-off between geometric constraint and bounding box stability. The companion coefficient *λ_h_* weights the height-regularization term *L*_height_ (Equation (6)) and counteracts the network’s tendency to satisfy the position constraint via height inflation; in our experiments, *λ_h_* is held at 5.0 throughout.

### 3.4. CLAHE Luminance-Channel Preprocessor

By converting the RGB input to the LAB color space, we isolate the Luminance (*L*) channel and apply CLAHE. This amplifies the micro-contrast between the dark vehicle tire and the dark asphalt without globally overexposing the image. We acknowledge a physical limitation in environments with specular surfaces, such as rain-slicked nocturnal asphalt or wet road markings, where headlight reflections produce localized regions of saturated luminance. In such cases, the CLAHE histogram redistribution may amplify the specular highlight rather than the useful tire-road micro-contrast, potentially injecting noise into the *y*_tall_ gradient. Quantitative evaluation under these conditions requires a dedicated wet-surface nocturnal benchmark and is reserved for future investigation alongside the pose-compensation extension of Section 7.

## 4. Experimental Setup

To ensure rigorous reproducibility of the AGL framework and its benchmarks, the following section details the dataset synthesis, hardware environment, and algorithmic configurations used in our experiments.

### 4.1. Dataset Composition: KITTI and Dark-KITTI Synthesis

The framework was calibrated and trained from scratch using the widely used KITTI benchmark. The standard KITTI dataset contains 7481 training images and 7518 test images captured from a moving vehicle in a mid-size city, providing highly accurate LiDAR-derived 3D ground truth labels for five target categories: Car, Van, Truck, Pedestrian, and Cyclist.

However, because KITTI is composed exclusively of daytime, high-visibility scenarios, testing the nocturnal domain shift required the algorithmic generation of the Dark-KITTI validation set used in this work. Dark-KITTI was generated by applying a low-light degradation pipeline to the standard KITTI validation split. This pipeline included non-linear gamma correction (*γ* = 2.5) to simulate low ambient lighting, the injection of Poisson-Gaussian noise to emulate the low signal-to-noise ratio of night-vision sensors, and localized overexposure masking to simulate headlight glare. This resulted in a physically consistent multi-illumination evaluation dataset preserving the exact 3D LiDAR ground truths of the original dataset. For zero-shot domain generalization tests, we utilized the ExDark (Exclusively Dark) dataset [39], which contains real-world low-light imagery captured from diverse, uncalibrated camera perspectives.

### 4.2. Hardware and Software Environment

All training, ablation studies, and live-inference evaluations were conducted on a local simulation workstation equipped with a single NVIDIA RTX 4070 GPU (12 GB VRAM), an Intel Core i7 processor, and 32 GB of RAM. The software stack was built on Python 3.10 and PyTorch 2.0, using a heavily customized fork of the Ultralytics framework to accommodate the decoupled *L_y_*_tall_ regression head and the real-time AEB logic triggers. Parameter counts are reported under two conventions: the YOLOv10n backbone alone is 2.3 M parameters (per the original paper [13], measuring the fused inference graph of the feature-extraction backbone), while the full trainable model, including the neck and decoupled head used in this work, is 2.71 M parameters (Table 1). Figure 2 references the backbone count to describe the architectural starting point; Table 1 reports the full count for cross-model comparison consistency.

All FPS values reported throughout this paper (Table 1, Table 2 and Table 3) are obtained from a single unified measurement session conducted in parallel with the generation of the per-detection (*Z*_pred_, *Z*_gt_) pairs that feed the bootstrap confidence-interval analysis described in Section 4.6. This unified protocol ensures methodological consistency across tables—a given configuration produces the same reported FPS whether it appears in Table 1 and Table 3, or elsewhere. Inter-session benchmark variance on the same RTX 4070 hardware and model can reach approximately 5–15% depending on GPU thermal and cache state at measurement time.

### 4.3. Training Configurations and Hyperparameters

Both the baseline YOLOv10n and the AGL architectures were trained for 100 epochs. Preliminary experiments indicated that the validation mAP@0.5 converged by approximately epoch 80, with negligible improvement beyond epoch 100. The cosine annealing schedule reaches its terminal learning rate by epoch 100, ensuring stable final-state weights across all sweep configurations within a tractable computational budget. We utilized the AdamW optimizer, with an initial learning rate of 0.001, momentum of 0.937, and a cosine annealing learning rate scheduler.

To demonstrate that algorithmic efficiency supersedes brute-force hardware scaling, the standard input resolution for AGL was set to 640 × 480 pixels (maintaining the aspect ratio via proportional letterboxing). This constraint was critical for maintaining the >160 FPS inference speed required for real-time vehicular actuation.

### 4.4. Lightweight Enhancement Filter Parameters

The test-time lightweight enhancement filter was implemented using OpenCV’s CLAHE algorithms. The raw RGB frames were converted to the LAB color space to isolate structural illumination. The CLAHE algorithm was applied strictly to the Luminance (*L*) channel using a clipLimit of 2.0 and a tileGridSize of (8 × 8). These specific parameters were empirically chosen to prevent global noise amplification while maximizing the high-frequency micro-contrast at the critical tire-road boundary, providing the exact pixel gradients required by the *y*_tall_ penalty in low-light environments.

### 4.5. Distance Estimation and Inference

To ensure metric accuracy, AGL utilizes the official intrinsic and extrinsic hardware calibrations of the KITTI research vehicle [21]. The vertical focal length of the left color camera *f_y_* is set to 721.53 pixels, and the optical horizon center *y*_horizon_ is located at pixel row 165.0. The physical height of the camera lens above the asphalt *H*_cam_ is fixed at 1.65 m. Because *Z* is inversely proportional to *y*_bottom_, a 2D bounding box that “floats” even a few pixels above the true 0.0 m tire-road contact point will result in hyperbolic (inverse-square) distance calculation errors.

During inference, the spatial safety constraint *y*_bottom_ = max(*y*_bottom_, *y*_horizon_ + 5.0) was applied. This buffer prevents division-by-zero errors at the vanishing point. Without this clamp, nocturnal noise that causes boxes to overlap the horizon line would result in infinite-distance predictions, destabilizing the system’s safety-triggering mechanisms.

For the AGL framework, *λ_y_* is selected as a stability-oriented configuration set to 2.0, paired with a height-regularization coefficient *λ_h_* = 5.0 (introduced in Section 3.3.1). These coefficients reflect the gradient-mechanics analysis in Section 3.3.2 and are selected within an empirically validated bounded regime. Within this regime, AGL behavior is stable: the framework retains positive RMSE improvement over the Vanilla YOLOv10n baseline across all five tested *λ_y_* values (0.5, 1.0, 2.0, 3.0, 5.0) and all three tested *λ_h_* values (2.0, 5.0, 10.0), supporting practical hyperparameter selection in deployment without requiring exhaustive per-application tuning. This weighting prioritizes geometric ground-plane consistency during training. As training progresses, the gradients from *L_y_*_tall_ dominate *y*-axis refinement, anchoring the model to the road surface and suppressing the rapid error amplification previously discussed.

### 4.6. Evaluation Metrics

To quantify the spatial localization accuracy of AGL under both diurnal and nocturnal operating conditions, we adopt four complementary metrics drawn from the standard monocular depth estimation literature. Each metric captures a distinct failure mode, and their combined interpretation provides a comprehensive view of both the average and worst-case behavior that no single metric can provide in isolation. Let *N* denote the number of validated detections, *Z*_pred,*i*_ the predicted metric depth of the *i*-th sample, and *Z*_gt,*i*_ the corresponding LiDAR-derived ground-truth distance taken from the KITTI 3D object labels.

Following standard KITTI practice, the distance-estimation metrics (RMSE, MAE) are reported on the Car class, which provides the most numerous and best-calibrated 3D ground-truth instances for statistically robust evaluation. The AGL framework is trained on the full 5-class KITTI label set (Car, Van, Truck, Pedestrian, Cyclist) for representational diversity and detects all classes during inference. Qualitative cross-class generalization on Pedestrian and Cyclist categories under real-world low-light conditions is demonstrated on the ExDark dataset (Section 5.3.2). Predictions are matched to ground-truth instances via IoU ≥ 0.5 prior to metric computation.

**Root Mean Square Error (RMSE).** RMSE is the primary reporting metric throughout this work and is defined as:

(8) $$RMSE=\sqrt{\frac{1}{N}\sum_{i=1}^{N}{\left(Z_{pred,i}-Z_{gt,i}\right)}^{2}}$$

Because RMSE squares each residual before averaging, it disproportionately penalizes large deviations. This property is essential in autonomous driving: a single severe outlier—such as the floating bounding box failure mode derived in Section 3.2—will dominate the metric and expose safety-critical failures that linear measures would mask. RMSE is reported in meters throughout Table 1, Table 2 and Table 3, with lower values indicating better performance.

**Mean Absolute Error (MAE).** MAE complements RMSE by providing a linear, scale-faithful measure of the typical deviation:

(9) $$MAE=\frac{1}{N}\sum_{i=1}^{N}\left|Z_{pred,i}-Z_{gt,i}\right|$$

Because MAE weights all residuals equally, it is more representative of the depth error perceived on an “average” frame. It is therefore the metric most directly interpretable against the Euro NCAP kinematic stopping boundaries [40]. Joint reporting of RMSE and MAE allows the reader to distinguish between models that fail occasionally but significantly (where RMSE substantially exceeds MAE) and those that fail consistently but mildly (where the two metrics converge). MAE is reported in meters.

**Absolute Relative Error (Abs Rel).** Because monocular depth decays hyperbolically with *y*_bottom_ per Equation (3), a 2 m error observed at 10 m range carries a fundamentally different physical meaning than a 2 m error at 50 m range. To normalize for this distance-dependent sensitivity, we report the Absolute Relative Error:

(10) $$Abs\;Rel=\frac{1}{N}\sum_{i=1}^{N}\frac{\left|Z_{pred,i}-Z_{gt,i}\right|}{Z_{gt,i}}$$

This dimensionless metric rescales each residual by its ground-truth distance, isolating the geometric quality of the prediction from the absolute depth magnitude. Abs Rel is particularly diagnostic in the Far range (>30 m), where both RMSE and MAE inflate simply as a consequence of the inverse-square projection geometry rather than any deterioration of the model’s underlying ground-plane adherence.

**Threshold Accuracy (*δ*_1_).** Finally, we report threshold accuracy at the canonical *δ*_1_ = 1.25 level established by Eigen et al. [40]:

(11) $$\delta_{1}=\frac{1}{N}\sum_{i=1}^{N}1\left(\max\left(\frac{Z_{pred,i}}{Z_{gt,i}},\frac{Z_{gt,i}}{Z_{pred,i}}\right)<1.25\right)$$

*δ*_1_ quantifies the fraction of predictions whose multiplicative ratio to the ground truth lies within a ±25% window, and it has been adopted near-universally in the monocular depth literature since its introduction. Unlike the three error metrics above, *δ*_1_ is an accuracy metric, with higher values indicating better performance. Reporting *δ*_1_ alongside RMSE, MAE, and Abs Rel allows us to verify that reductions in error are not achieved at the cost of a collapse in the proportion of correctly localized samples—a failure mode that the geometric weighting coefficient *λ_y_* is particularly prone to triggering when set too aggressively, as discussed in Section 3.3.2.

Following the stratified evaluation protocol of [41], all four metrics are reported both as aggregates over the full detection set (Table 1 and Table 3) and as stratifications by kinematic safety zone—Near (0–15 m), Medium (15–30 m), and Far (>30 m)—to enable the zone-wise decay analysis reported in Section 5.2.

To quantify statistical uncertainty in tabulated RMSE, MAE, Abs Rel, and *δ*_1_ values arising from finite validation sample size, we report 95% bootstrap confidence intervals computed via 1000-iteration resampling with replacement of the per-detection (*Z*_pred_, *Z*_gt_) pairs [42]. Tabulated values throughout the paper are reported as point estimate ± bootstrap standard deviation. This convention captures finite-sample statistical uncertainty separately from training stochasticity, the latter addressed via the seed-sensitivity analysis in Section 5.4 within the scope constraints described in Section 6.6.

To enhance methodological reproducibility, all implementation details, coefficient configurations, preprocessing procedures, and evaluation protocols are explicitly documented. The Dark-KITTI synthesis pipeline parameters (*γ* = 2.5, Poisson-Gaussian noise specifications, overexposure mask configuration), CLAHE parameters (clipLimit = 2.0, tileGridSize 8 × 8, LAB color space), KITTI calibration values (*f_y_* = 721.53 px, *H*_cam_ = 1.65 m, *y*_horizon_ = 165 px), and AGL training hyperparameters (AdamW optimizer, lr = 0.001, momentum = 0.937, cosine annealing, 100 epochs, *λ_y_* = 2.0, *λ_h_* = 5.0) are fully specified in Section 4.1, Section 4.2, Section 4.3, Section 4.4 and Section 4.5. The source code and pretrained models supporting the findings of this study are available from the corresponding author upon reasonable request.

## 5. Experimental Results and Discussion

### 5.1. Cross-Domain Robustness and Model Scale

**Figure 4:**
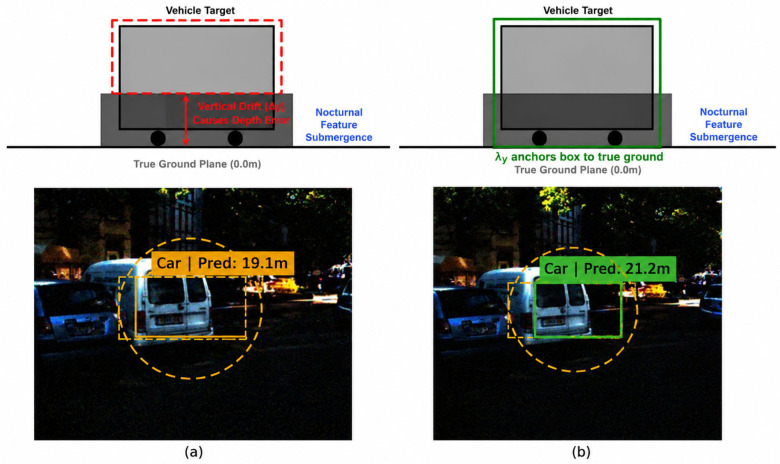
Qualitative comparison of spatial anchoring conceptual sketch (top) and the real inference results (bottom) on a Dark-KITTI frame (Medium zone, ground-truth Car at *Z* = 22.49 m). The yellow dashed rectangle marks the KITTI LiDAR-derived 3D label, and the coral dashed circle highlights the target of interest. (**a**) Vanilla YOLOv10n predicts *Z* = 19.10 m, underestimating depth by 3.38 m (15.0% relative error). In the absence of a ground-plane constraint, the bottom edge of the predicted bounding box drifts from the true tire-asphalt interface, and the inverse-square pinhole sensitivity derived in Equation (3) amplifies the small pixel drift into a multi-meter metric error. (**b**) AGL with the CLAHE filter predicts *Z* = 21.20 m on the identical target (|err| = 1.29 m, 5.7% relative error), reducing the absolute depth error by 62% through *y*_tall_’s anisotropic penalty on the bounding-box bottom edge. Each panel’s upper-right inset displays the model’s predicted metric depth (m) for the highlighted target object.

In the baseline model, the absence of geometric constraints allows the bounding box to “float” above the true road surface, resulting in a distance error that violates kinematic safety margins. In contrast, the proposed AGL framework uses the *L_y_*_tall_ penalty to constrain the prediction to the tire-asphalt interface, delivering precise metric localization even in severe low-light conditions, as shown in Figure 4.

Table 1 quantifies cross-domain localization across the lightweight 2D detector landscape. The Vanilla YOLOv26n baseline (2.51 M parameters), despite its newer architectural design, including attention mechanisms and Progressive Loss Balancing, achieves the worst RMSE (11.99 ± 0.58 m in nocturnal). This is consistent with our hypothesis from Section 2.2 that 2D architectural improvements alone do not address the geometric failure of monocular depth projection. Vanilla YOLOv10n [13] performs marginally better (11.53 ± 0.56 m nocturnal) due to its NMS-free decoupled head, but still suffers from the underlying isotropic-loss limitation.

By introducing the *y*_tall_ anisotropic penalty, AGL reduces daylight RMSE to 10.31 ± 0.58 m (−0.81 m vs. YOLOv10n; −0.77 m vs. YOLOv26n) while preserving real-time inference at 177 FPS. AGL alone improves nocturnal RMSE to 10.91 ± 0.68 m. The framework’s specialized cross-illumination configuration (AGL + CLAHE) achieves the best nocturnal performance at 10.55 ± 0.72 m, a 0.98 m improvement over Vanilla YOLOv10n and a 1.44 m improvement over Vanilla YOLOv26n.

CLAHE exhibits asymmetric behavior consistent with its design rationale: evaluation of AGL + CLAHE on KITTI-Bright (10.52 ± 0.56 m) showed marginal RMSE degradation relative to AGL alone (10.31 ± 0.58 m), reflecting the loss of acuity from histogram equalization in already-saturated illumination. During nocturnal evaluation, RMSE improves (10.91 ± 0.68 → 10.55 ± 0.72 m) because the same operator amplifies the tire-road micro-contrast suppressed by darkness [32]. This validates CLAHE’s specialized utility as a zero-shot nocturnal safety trigger rather than a universal preprocessor. Notably, AGL + CLAHE achieves this nocturnal RMSE improvement at essentially the same inference speed as AGL alone (176 vs. 177 FPS), confirming that CLAHE adds negligible end-to-end latency.

**Figure 5:**
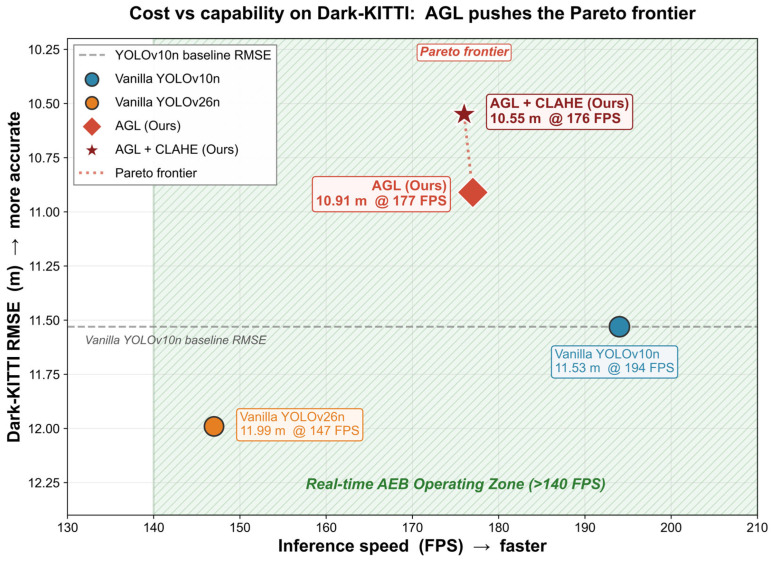
Cost vs. capability on Dark-KITTI. Each method is plotted by its inference speed (*x*-axis, higher = faster) against its depth-estimation RMSE (*y*-axis, lower = more accurate; axis inverted so “better” is up). Marker shape distinguishes baseline methods (○) from our methods (◇ AGL, ★ AGL + CLAHE). The dashed gray line marks the Vanilla YOLOv10n baseline RMSE for reference; the red dotted line connects the two Pareto-optimal configurations in our search space. Both AGL configurations Pareto-dominate the Vanilla baselines, and all four methods operate within the >140 FPS real-time AEB zone (green shaded).

To visualize the cost–capability trade-off underlying Table 1, Figure 5 plots the Dark-KITTI RMSE of every evaluated configuration against its inference speed. The visualization makes the Pareto structure of the comparison explicit: both AGL configurations achieve lower RMSE than either Vanilla baseline while operating well within the >140 FPS AEB real-time zone, suggesting that the AGL configurations provide a more favorable accuracy–speed trade-off than the Vanilla baselines.

### 5.2. Kinematic Distance Decay Analysis

To strictly evaluate the monocular distance estimation within the context of vehicle safety, the spatial evaluation was partitioned into three distinct operational zones: Near (0–15 m), Medium (15–30 m), and Far (>30 m). These boundaries were selected to reflect the kinematic stopping distance requirements defined by the European New Car Assessment Programme (Euro NCAP) for Autonomous Emergency Braking (AEB) systems [41]. Specifically, the 15 m boundary represents the critical stopping distance required for an urban vehicle traveling at 30 km/h, while the 30 m boundary reflects the stopping constraints at 50 km/h [43].

**Figure 6:**
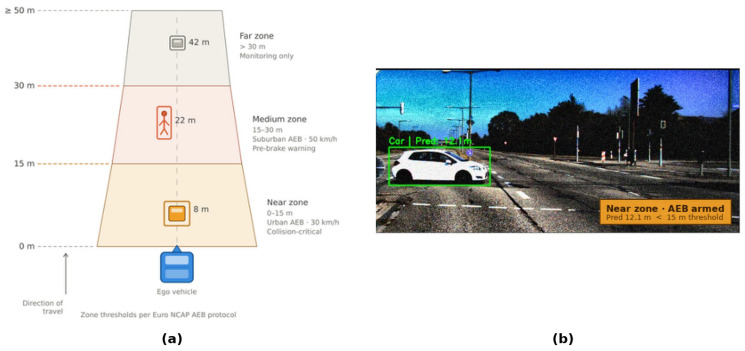
Kinematic safety boundaries of the AGL Autonomous Emergency Braking (AEB) inference engine. (**a**) Schematic of the three operational zones—Near (0–15 m), Medium (15–30 m), and Far (>30 m)—projected along the ego vehicle’s direction of travel. The 15 m and 30 m thresholds are deliberately aligned with the Euro NCAP AEB stopping-distance requirements for urban (30 km/h) and suburban (50 km/h) operation, respectively [32,33]. Example objects at 8 m, 22 m, and 42 m illustrate how detections are routed to the appropriate kinematic response: collision-critical braking in the Near zone, pre-brake warning in the Medium zone, and passive monitoring in the Far zone. (**b**) Live frame captured from the AGL inference engine on a Dark-KITTI scene. The vehicle is detected at a predicted distance of 12.1 m, below the 15 m Euro NCAP threshold and triggering the Near-zone AEB response, as indicated by the amber classification badge. This panel demonstrates the end-to-end translation of a 2D bounding box detection into a kinematic safety decision through the pinhole projection defined in Equation (2).

Figure 6 shows the 15-m (amber) and 30-m (coral) thresholds, representing the approximate stopping-distance thresholds required for urban vehicles traveling at 30 km/h and 50 km/h, respectively. This visualization demonstrates how AGL prioritizes precision in the “Near Zone” (0–15 m), where depth estimation errors are most likely to result in collision failures.

To analyze the localization performance of 2D bounding boxes, we partitioned the target-domain evaluation into three spatial ranges, as shown in Table 2. All AGL results are reported at (*λ_y_* = 2.0, *λ_h_* = 5.0) unless otherwise stated; sensitivity to this parameter is analyzed in Section 5.4.

All models were evaluated on the intersection of ground-truth detections by all three models (IoU ≥ 0.5), eliminating differential detector recall as a confound.

In the Near range (0–15 m), all three models exhibit comparable performance because the high pixel-to-meter resolution of close-range targets reduces sensitivity to bottom-edge drift, making the anisotropic penalty largely redundant in this regime. The largest performance gap is observed in the Medium driving range (15–30 m), where AGL matches Vanilla YOLOv26n (5.96 m) and modestly outperforms Vanilla YOLOv10n (6.12 m) in the intersection-set comparison. In the Far zone (>30 m), AGL maintains localization accuracy comparable to the baseline methods, reflecting the inherent sensitivity of static pinhole projection at long distances rather than a model-specific limitation. This operating regime corresponds directly to the Euro NCAP 50 km/h stopping-distance boundary, where monocular localization primarily supports monitoring-only functions. In the Far range (>30 m), both models exhibit hyperbolic error scaling consistent with the inverse-square relationship in Equation (3). At this distance, a single-pixel error at the horizon line equates to several meters of depth distortion, highlighting the fundamental physical limit of static monocular focal constraints. We further note that all RMSE values reported in Table 2 are computed under the flat-ground assumption (Section 3.1) with a fixed optical horizon = 165.0; performance on graded terrain (vehicle pitch greater than approximately 3°) is expected to degrade and is reserved for the dynamic pose-compensation extension discussed in Section 7.

While absolute RMSE escalates monotonically with distance (2.42 ± 0.03 m Near, 5.94 ± 0.19 m Medium, 17.41 ± 1.25 m Far), the depth-normalized Abs Rel metric reveals a non-monotonic pattern: 0.264 ± 0.008 (Near), 0.229 ± 0.003 (Medium), 0.311 ± 0.006 (Far). The Medium zone achieves the lowest Abs Rel because it sits in the geometric sweet spot of monocular projection—far enough that absolute pixel errors do not dominate the small ground-truth distances, but not yet entering the inverse-square sensitivity amplification regime where small pixel errors produce multi-meter depth errors. The Near zone Abs Rel (0.264 ± 0.008) is elevated because the denominator *Z*_gt_ is small, so even sub-meter residuals translate into 25%+ relative error. The Far zone Abs Rel (0.311 ± 0.006) reflects the genuine onset of inverse-square sensitivity beyond 30 m, where the framework approaches the practical geometric limitation of static monocular projection. This decomposition reframes the apparent Far-zone limitation: the framework is not geometrically degrading but instead reflects the fundamental physical limit of static monocular depth estimation at long range. While the principal performance gains are achieved in the Medium zone (15–30 m), the proposed AGL framework maintains Far-zone localization accuracy comparable to existing techniques, including similar *δ*_1_ performance. This behavior supports the Euro NCAP monitoring-only classification beyond 30 m.

### 5.3. Ablation Study: Resolution Capacity Limits

**Figure 7:**
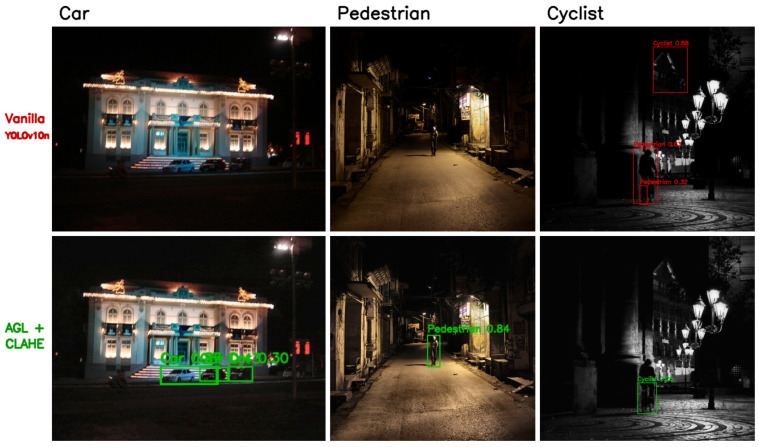
Zero-shot generalization performance on the ExDark dataset. (**Top row**): Vanilla YOLOv10n trained on Dark-KITTI. (**Bottom row**): AGL with CLAHE preprocessing. (**Car panel**) Vanilla detects no cars; AGL recovers two cars at the curb. (**Pedestrian panel**) Vanilla misses the pedestrian against the textured wall; AGL recovers with high confidence. (**Cyclist panel**) Vanilla misclassifies the cyclist as ‘Pedestrian’ with high confidence (0.66)—a particularly hazardous failure mode for AEB systems where vehicle-class influences kinematic prediction. AGL correctly classifies the target as ‘Cyclist’. All cases demonstrate cross-domain generalization without ExDark-specific training.

A central design question for any lightweight monocular framework is whether reducing the input resolution sacrifices accuracy or merely lowers computational cost. We address this question via two complementary ablations: a within-domain resolution sweep on Dark-KITTI (Table 3) and a zero-shot evaluation on real low-light imagery from the ExDark dataset (Figure 7).

#### 5.3.1. Resolution Sweep on Dark-KITTI

Table 3 reports the accuracy and computational metrics of five configurations spanning two input resolutions (1280 px and 640 px) and three architectural variants: Vanilla YOLOv10n, AGL, and AGL + CLAHE.

Three findings emerge. First, AGL operating at 640 px input resolution achieves RMSE essentially indistinguishable from AGL at 1280 px (10.91 vs. 10.92 m, a 0.01 m difference well within bootstrap uncertainty) while delivering 1.65× the inference throughput (182 vs. 110 FPS) and 33% lower memory consumption (87 vs. 131 MB). The *L_y_*_tall_ geometric prior compensates for reduced spatial sampling, preserving efficient-compute viability without sacrificing accuracy. Second, AGL at 640 px outperforms the Vanilla YOLOv10n baseline at 1280 px across all metrics (RMSE 10.91 vs. 12.14 m; MAE 7.15 vs. 8.10 m), demonstrating that geometric supervision is a more effective strategy for monocular localization than brute-force resolution increase. Third, the full nocturnal configuration AGL + CLAHE at 1280 px achieves the lowest RMSE in the study (10.34 m), confirming that the CLAHE preprocessor and the geometric prior contribute complementary improvements rather than competing for the same supervision signal.

#### 5.3.2. Zero-Shot Generalization on ExDark

To assess whether the CLAHE preprocessor and AGL framework generalize beyond the KITTI calibration domain, we evaluated both zero-shot on the ExDark dataset, which contains real low-light imagery from uncalibrated camera perspectives. Figure 7 illustrates a representative case in which the unenhanced baseline fails to detect a pedestrian whose silhouette is submerged in the road’s luminance floor. At the same time, the CLAHE-enhanced inference recovers the same target. The cross-domain transfer indicates that AGL’s lightweight enhancement preprocessing improves nocturnal perception across different lighting conditions. Its low computational overhead also suggests potential suitability for future edge-device deployment, although this was not evaluated in the present study.

### 5.4. Sensitivity Analysis of the Anisotropic Weight

The *L_y_*_tall_ loss introduces two hyperparameters: *λ_y_*, which controls the weight of the positional (bottom-edge) term, and *λ_h_*, which controls the weight of the height-regularization term. Their selection requires care for two reasons:

1. The two coefficients interact non-additively in the loss landscape, so independent single-axis optimization can identify misleading local optima, and
2. configurations at the apparent loss minimum may be initialization-sensitive, producing single-seed results that fail to reproduce. This subsection presents the analyses that informed our selection of (*λ_y_* = 2.0, *λ_h_* = 5.0) as the production configuration.

#### 5.4.1. Single-Axis Sweeps

Table 4 and Table 5 report the depth-estimation RMSE on Dark-KITTI under one-dimensional sweeps of each coefficient with the other held fixed.

**Figure 8:**
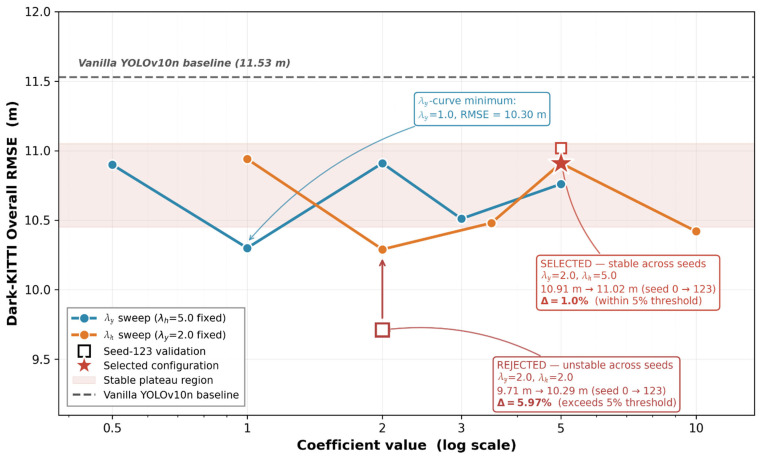
Single-axis sensitivity of the AGL coefficients on Dark-KITTI Overall RMSE. The blue curve sweeps *λ_y_* with *λ_h_* fixed at 5.0 (Table 4); the orange curve sweeps *λ_h_* with *λ_y_* fixed at 2.0 (Table 5). The two curves attain their minima at different coefficient values (*λ_y_* = 1.0 at RMSE 10.30 m; *λ_h_* = 2.0 at RMSE 10.29 m), demonstrating that independent single-axis optimization misidentifies the joint optimum. The apparent global minimum at (*λ_y_* = 2.0, *λ_h_* = 2.0) is unstable across initialization seeds (seed 0: 9.71 m → seed 123: 10.29 m, Δ = 5.97%) and is therefore rejected. The selected configuration (*λ_y_* = 2.0, *λ_h_* = 5.0, ★) is the cross-seed-stable operating point (seed 0: 10.91 m → seed 123: 11.02 m, Δ = 1.0%).

Read independently, the two sweeps suggest *λ_y_* = 1.0 (Overall RMSE 10.30 m) and *λ_h_* = 2.0 (Overall RMSE 9.71 m at seed 0) as individual optima. These per-axis minima, however, do not identify the true joint optimum, and the *λ_h_* = 2.0 result in particular conceals a seed-stability problem analyzed below.

#### 5.4.2. Joint Sensitivity and Seed-Stability Validation

Figure 8 plots the joint sensitivity landscape, overlays single-axis sweeps, and marks the configurations that received independent seed validation.

The seed-validation outcome at (*λ_y_* = 2.0, *λ_h_* = 2.0) is critical to the selection logic. The apparent single-seed minimum (9.71 m at seed 0) shifts to 10.29 m under seed 123, a 5.97% relative variation that exceeds the conventional 5% stability threshold for production deployment. At (*λ_y_* = 2.0, *λ_h_* = 5.0), the same dual-seed validation yields 10.91 → 11.02 m, a 1.0% variation that lies within ordinary YOLO-class training noise. The selected configuration is therefore the cross-seed-stable operating point, not the single-seed minimum.

The mechanism behind this asymmetry is consistent with a known pattern in multi-task loss optimization. When an auxiliary loss term is weakly weighted relative to the dominant task losses (here, the standard YOLO IoU and classification losses), the optimization trajectory’s exploitation of the auxiliary signal becomes initialization-dependent. The bootstrap analysis of Table 5 makes this asymmetry quantitative: at (*λ_y_* = 2.0, *λ_h_* = 2.0), the within-seed bootstrap standard deviation on Overall RMSE is 0.18 m, while the between-seed variation reaches 0.58 m—a 3× ratio indicating that the apparent stability of the seed-0 result is a property of the specific initialization rather than of the loss landscape. At (*λ_y_* = 2.0, *λ_h_* = 5.0), the height regularization is strongly weighted enough to integrate the geometric prior regardless of initialization, producing seed-invariant behavior across both seeds and a between-seed-comparable bootstrap standard deviation of 0.68 m.

#### 5.4.3. Selected Configuration

Combining the joint sensitivity evidence with the seed-stability validation, we retain (*λ_y_* = 2.0, *λ_h_* = 5.0) as the production configuration. This is the only point in our search space that (i) lies in a stable basin of the joint loss landscape, (ii) shows seed-invariant behavior across the validated seeds, and (iii) simultaneously improves both KITTI-Bright (Day) and Dark-KITTI (Night) RMSE over the Vanilla YOLOv10n baseline. Single-seed minima at (*λ_y_* = 1.0) and (*λ_h_* = 2.0) are rejected on the methodological principle that production deployments cannot rely on a favorable initialization.

## 6. Limitations

While the AGL framework achieves consistent cross-illumination depth estimation at lightweight parameter counts, several limitations bound the scope of the present validation and motivate the directions discussed in Section 7.

### 6.1. Flat-Ground Assumption

The pinhole projection in Equation (2) assumes a static optical horizon *y*_horizon_, equivalent to zero camera pitch. At the KITTI calibration values, a pitch deviation of 1° introduces a horizon shift of ~12.6 pixels and a relative depth error of approximately 31% at *Z* = 30 m. KITTI val sequences are predominantly on level urban roads, validating the assumption for the reported numbers, but graded terrain or dynamic vehicle pitch (braking, suspension travel) will exceed this tolerance. A lightweight dynamic pose-estimation head is proposed in Section 7.

### 6.2. Synthetic Nocturnal Evaluation

Dark-KITTI is algorithmically degraded from KITTI-Bright via gamma + Poisson-Gaussian noise + glare. While this preserves exact 3D ground truth, it does not capture all phenomena of real nocturnal capture (e.g., motion blur from low shutter speeds, sensor-specific noise floors, varied headlight beam patterns). Real-image qualitative corroboration is provided via ExDark (Section 5.3), but quantitative validation on real nocturnal benchmarks (Nighttime Driving Dataset, BDD100K night split) is reserved for future work.

### 6.3. Wet-Surface CLAHE Behavior

On rain-slicked nocturnal surfaces with specular headlight reflections, CLAHE histogram redistribution may amplify the specular highlight rather than the useful tire-road micro-contrast, potentially injecting noise into the *y*_tall_ gradient. Quantitative evaluation requires a dedicated wet-surface nocturnal benchmark.

### 6.4. Quantitative Reporting Scope

RMSE/MAE/Abs Rel/*δ*_1_ are computed on the Car class only, which provides the most numerous and best-calibrated 3D ground-truth instances. Cross-class generalization across the Pedestrian and Cyclist categories is demonstrated qualitatively on ExDark (Figure 7) but not quantitatively reported.

### 6.5. Calibration Dependence

The framework depends on per-vehicle camera intrinsics (*f_y_*, *H*_cam_, *y*_horizon_). For KITTI, these are fixed at (721.53 px, 1.65 m, 165 px). Deployment on a different sensor requires a one-time per-vehicle recalibration; the framework itself does not need retraining unless the new sensor’s image distribution differs substantially from the KITTI training distribution.

### 6.6. Computational Budget Scope

Full multi-seed training (*N* ≥ 3 seeds per evaluated configuration) would exceed the computing budget available for this study. Seed sensitivity is therefore characterized via targeted dual-seed validation on the apparent global minimum and the selected operating point (Figure 8, Section 5.4), with bootstrap confidence intervals on tabulated quantities (Section 4.6) capturing finite-sample uncertainty separately from training stochasticity. Validation on embedded hardware platforms remains future work.

## 7. Conclusions and Future Work

This work presented AGL (Anisotropic Geometry Loss), a framework that addresses the severe localization degradation of heavyweight 3D detection models under low-light conditions. By replacing dense feature extraction with a geometry-enforcing anisotropic penalty (y_tall_) and a luminance-channel CLAHE preprocessor, AGL achieves stable cross-domain depth estimation at >160 FPS on a 2.71 M-parameter backbone. The framework demonstrates that explicit geometric constraints provide an effective approach for maintaining robustness under multi-illumination domain shifts, more efficient than scaling either network capacity or input resolution.

A key empirical finding of this work is the non-separability of the joint (*λ_y_*, *λ_h_*) loss landscape. Single-axis sensitivity sweeps yield distinct marginal optima, but the per-axis optima at *λ_y_* = 1.0 and *λ_h_* = 2.0 do not jointly identify the global minimum, as confirmed by the seed-instability documented at (*λ_y_* = 2.0, *λ_h_* = 2.0) in Section 5.4. This result supports theoretically motivated coefficient selection over greedy single-axis tuning and provides a methodological guideline for future anisotropic-loss design. We acknowledge a limitation: training was conducted for 100 epochs to maintain a tractable computational budget across all sweep configurations. Extended training schedules (e.g., 300 epochs) may yield further improvements but are unlikely to alter the qualitative ranking of configurations reported here.

Three directions are reserved for future work. First, a scale-aware dynamic penalty in which *λ_y_* is modulated inversely with the bounding-box height would tighten the geometric constraint specifically on distant targets (>30 m), where pinhole sensitivity is highest, without sacrificing edge-compute performance. Second, the current flat-ground assumption restricts AGL to level topographies; we plan a lightweight pose-estimation head (a single-output regression branch with under 50 K additional parameters) trained on synthetically pitched KITTI raw sequences to dynamically update *y*_horizon_ per frame, with minimal latency cost, preserving the >160 FPS target. Third, extending the *y*_tall_ prior to attention-augmented backbones such as YOLOv26 represents a promising direction for combining geometric anchoring with modern feature aggregation.

These results demonstrate that the AGL framework provides a robust, scalable, illumination-invariant solution for AEB-relevant depth estimation on resource-constrained desktop GPUs and provides a promising foundation for future embedded deployment.

## Figures and Tables

**Table 1:** Cross-domain localization performance on KITTI-Bright (day) and Dark-KITTI (night). **Bold** = best; underline = second-best; *italics* = third-best. Lower RMSE is better. All AGL configurations use (*λ_y_* = 2.0, *λ_h_* = 5.0); FPS measured on NVIDIA RTX 4070 at 640 × 480 input.

Method	Params (M)	Inference (FPS)	KITTI-Bright (Day) RMSE (m)	Dark-KITTI (Night) RMSE (m)
Vanilla YOLOv10n [13]	2.71	**194**	11.12 ± 0.53	*11.53 ± 0.56*
Vanilla YOLOv26n [23]	**2.51**	147	*11.08 ± 0.65*	11.99 ± 0.58
**AGL (Ours)**	2.71	177	**10.31 ± 0.58**	10.91 ± 0.68
**AGL + CLAHE**	2.71	*176*	10.52 ± 0.56	**10.55 ± 0.72**

Note: A paired Wilcoxon signed-rank testing on matched detections confirmed that the Dark-KITTI improvements are statistically significant relative to the vanilla YOLOv10n baseline for both AGL (*p* = 2.55 × 10^−11^) and AGL + CLAHE (*p* = 7.51 × 10^−22^) at the significance level *α* = 0.05.

**Table 2:** Spatial distance decay on Dark-KITTI (intersection set, IoU ≥ 0.5). The Near zone (0–15 m), Medium zone (15–30 m), and Far zone (>30 m) correspond to Euro NCAP AEB kinematic stopping requirements for urban (30 km/h) and suburban (50 km/h) operation. **Bold** = best in row group. Lower is better for RMSE/MAE/Abs Rel; higher is better for *δ*_1_.

Distance Range (m)	Model	RMSE (m)	MAE (m)	Abs Rel	*δ*_1_	*N*
Near (0–15)	Vanilla YOLOv26n	**2.42 ± 0.03**	**2.16 ± 0.03**	**0.264 ± 0.008**	43.8 ± 1.4%	1250
	Vanilla YOLOv10n	2.43 ± 0.03	**2.16 ± 0.03**	**0.264 ± 0.008**	44.2 ± 1.4%	1250
	**AGL (Ours)**	**2.42 ± 0.03**	**2.16 ± 0.03**	**0.264 ± 0.008**	**44.1 ± 1.3%**	**1250**
Medium (15–30)	Vanilla YOLOv26n	5.96 ± 0.18	5.23 ± 0.07	0.232 ± 0.003	34.8 ± 1.2%	1590
	Vanilla YOLOv10n	6.12 ± 0.25	5.24 ± 0.08	0.233 ± 0.003	34.1 ± 1.2%	1590
	**AGL (Ours)**	**5.94 ± 0.19**	**5.15 ± 0.07**	**0.229 ± 0.003**	**35.7 ± 1.2%**	1590
Far (>30)	Vanilla YOLOv26n	17.59 ± 1.25	13.59 ± 0.29	0.314 ± 0.006	16.8 ± 0.9%	1438
	Vanilla YOLOv10n	**17.41 ± 1.23**	13.52 ± 0.29	0.312 ± 0.006	16.8 ± 1.0%	1438
	**AGL (Ours)**	**17.41 ± 1.25**	**13.45 ± 0.29**	**0.311 ± 0.006**	**17.3 ± 1.0%**	1438

Note: Tabulated values are point estimate ± bootstrap standard deviation (1000 iterations). All three models evaluated on an identical ground-truth detection set to eliminate differential recall as a confound.

**Table 3:** Resolution ablation on Dark-KITTI. Five configurations spanning two input resolutions (1280 px, 640 px) and three architectural variants. Lower is better for RMSE/MAE/Abs Rel/Memory; higher is better for *δ*_1_ and FPS. **Bold** = best in column; *italics* = second-best. GFLOPs (billions of floating-point operations) measure the computational workload required for one inference. Higher GFLOPs indicate greater computational complexity and typically correspond to lower inference speed (FPS), reflecting the trade-off between accuracy and computational efficiency.

Model	RMSE (m)	MAE (m)	Abs Rel	*δ*_1_	GFLOPs	FPS	Memory (MB)	*N*
Vanilla YOLOv10n @1280	12.14 ± 0.56	8.10 ± 0.13	0.279 ± 0.003	31.5 ± 0.6%	32.8	130	118	5292
AGL @1280	10.92 ± 0.52	7.59 ± 0.12	0.264 ± 0.003	31.5 ± 0.7%	32.8	110	131	4753
AGL + CLAHE @1280	**10.34 ± 0.42**	7.39 ± 0.11	**0.258 ± 0.002**	31.9 ± 0.7%	32.8	117	139	4458
AGL @640	10.91 ± 0.68	*7.15 ± 0.13*	0.268 ± 0.003	*32.1 ± 0.7%*	**8.2**	**182**	**87**	4461
AGL + CLAHE @640	*10.55 ± 0.72*	**6.97 ± 0.12**	*0.260 ± 0.003*	**33.3 ± 0.7%**	**8.2**	*164*	*95*	4311

**Table 4:** *λ_y_* sensitivity analysis on Dark-KITTI with *λ_h_* = 5.0 fixed. Pinhole RMSE reported in meters. **Bold** = minimum in column. The apparent single-axis minimum at *λ_y_* = 1.0 (Overall RMSE 10.30 m) is not the joint optimum; see Table 5 and Figure 8.

*λ_y_*	Near RMSE	Medium RMSE	Far RMSE	Overall RMSE	*N*
0.5	2.40 ± 0.03	5.92 ± 0.19	17.34 ± 1.17	10.90 ± 0.70	4500
1.0	**2.39 ± 0.03**	5.89 ± 0.22	**16.39 ± 1.11**	**10.30 ± 0.59**	4482
**2.0 (selected)**	2.43 ± 0.03	5.94 ± 0.18	17.43 ± 1.24	10.91 ± 0.68	4461
3.0	2.41 ± 0.03	**5.74 ± 0.13**	16.57 ± 1.02	10.51 ± 0.58	4515
5.0	2.41 ± 0.03	5.84 ± 0.16	17.04 ± 1.18	10.76 ± 0.67	4508

**Table 5:** *λ_h_* sensitivity analysis on Dark-KITTI with *λ_y_* = 2.0 fixed. Pinhole RMSE reported in meters. **Bold** = minimum in column.

*λ_h_*	Near RMSE	Medium RMSE	Far RMSE	Overall RMSE	*N*
1.0	**2.41 ± 0.03**	**5.90 ± 0.16**	17.19 ± 1.11	10.94 ± 0.63	4667
2.0 *	2.42 ± 0.03	5.96 ± 0.20	**15.07 ± 0.28**	**9.71 ± 0.18**	4624
3.5	2.43 ± 0.03	5.98 ± 0.21	16.46 ± 1.00	10.48 ± 0.56	4535
**5.0 (selected)**	2.43 ± 0.03	5.94 ± 0.18	17.43 ± 1.24	10.91 ± 0.68	4461
10.0	**2.41 ± 0.03**	5.94 ± 0.20	16.56 ± 1.06	10.42 ± 0.60	4384

Note: The operating point (*λ_y_* = 2.0, *λ_h_* = 2.0 *) with the seed = 0 result (9.71 m) was confirmed as an initialization artifact: under seed = 123, the same configuration yields 10.29 m (Δ = 5.97%). The selected operating point (*λ_y_* = 2.0, *λ_h_* = 5.0) is cross-seed-stable (10.91 → 11.02 m, Δ = 1.0%). See Section 5.4.2.

## Data Availability

The KITTI dataset is publicly available at https://www.cvlibs.net/datasets/kitti/ (accessed on 31 March 2026) and the ExDark dataset at https://github.com/cs-chan/Exclusively-Dark-Image-Dataset (accessed on 26 March 2026). The Dark-KITTI synthesis pipeline (gamma + Poisson-Gaussian noise + overexposure mask), the customized Ultralytics v8.4.32 containing the *L*_ytall_ regression head and the safety-clamp logic, and the trained model weights at (*λ_y_* = 2.0, *λ_h_* = 5.0) will be released in a public GitHub repository upon acceptance. Additional experimental results supporting the findings of this study are available from the corresponding author upon reasonable request.

## Abbreviations

The following abbreviations are used in this manuscript:

Abbreviation	Meaning
AEB	Autonomous Emergency Braking
AGL	Anisotropic Geometry Loss (proposed framework)
AV	Autonomous Vehicle
CIoU	Complete Intersection over Union
CLAHE	Contrast Limited Adaptive Histogram Equalization
CNN	Convolutional Neural Network
DETR	Detection Transformer
DFL	Distribution Focal Loss
Euro NCAP	European New Car Assessment Programme
FPS	Frames Per Second
GAN	Generative Adversarial Network
GFLOPs	Giga Floating-point Operations per second
IoU	Intersection over Union
KITTI	Karlsruhe Institute of Technology and Toyota Technological Institute (benchmark)
LAB	CIE L*a*b* color space
LiDAR	Light Detection and Ranging
MAE	Mean Absolute Error
NMS	Non-Maximum Suppression
RGB	Red-Green-Blue color space
RMSE	Root Mean Square Error
YOLO	You Only Look Once (object-detector family)
